# Assessing Natural Resource Use by Forest-Reliant Communities in Madagascar Using Functional Diversity and Functional Redundancy Metrics

**DOI:** 10.1371/journal.pone.0024107

**Published:** 2011-09-01

**Authors:** Kerry A. Brown, Dan F. B. Flynn, Nicola K. Abram, J. Carter Ingram, Steig E. Johnson, Patricia Wright

**Affiliations:** 1 School of Geography, Geology and the Environment, Kingston University London, Surrey, United Kingdom; 2 Department of Ecology, Evolution, and Environmental Biology, Columbia University, New York, New York, United States of America; 3 Durrell Institute of Conservation and Ecology (DICE), University of Kent, Canterbury, Kent, United Kingdom; 4 Wildlife Conservation Society, Bronx, New York, United States of America; 5 Department of Anthropology, University of Calgary, Calgary, Alberta, Canada; 6 Institute for the Conservation of Tropical Environments, Stony Brook University, Stony Brook, New York, United States of America; University of Montpellier II, France

## Abstract

Biodiversity plays an integral role in the livelihoods of subsistence-based forest-dwelling communities and as a consequence it is increasingly important to develop quantitative approaches that capture not only changes in taxonomic diversity, but also variation in natural resources and provisioning services. We apply a functional diversity metric originally developed for addressing questions in community ecology to assess utilitarian diversity of 56 forest plots in Madagascar. The use categories for utilitarian plants were determined using expert knowledge and household questionnaires. We used a null model approach to examine the utilitarian (functional) diversity and utilitarian redundancy present within ecological communities. Additionally, variables that might influence fluctuations in utilitarian diversity and redundancy—specifically number of felled trees, number of trails, basal area, canopy height, elevation, distance from village—were analyzed using Generalized Linear Models (GLMs). Eighteen of the 56 plots showed utilitarian diversity values significantly higher than expected. This result indicates that these habitats exhibited a low degree of utilitarian redundancy and were therefore comprised of plants with relatively distinct utilitarian properties. One implication of this finding is that minor losses in species richness may result in reductions in utilitarian diversity and redundancy, which may limit local residents' ability to switch between alternative choices. The GLM analysis showed that the most predictive model included basal area, canopy height and distance from village, which suggests that variation in utilitarian redundancy may be a result of local residents harvesting resources from the protected area. Our approach permits an assessment of the diversity of provisioning services available to local communities, offering unique insights that would not be possible using traditional taxonomic diversity measures. These analyses introduce another tool available to conservation biologists for assessing how future losses in biodiversity will lead to a reduction in natural resources and provisioning services from forests.

## Introduction

The livelihoods of rural, subsistence societies living in and around forests are intimately connected to the natural resources that can be procured from these ecosystems [1], [2]. The diversity of wild plants within forests supply people with a range of services, such as wood for fuel, timber for construction, as well as numerous non-timber forest products (NTFPs) for food, weaving and medicines [3]. Access to these resources is necessary for sustaining livelihood practices and preserving important cultural, commercial, and spiritual activities [4], [5], [6]. Over time, these natural resources may become depleted through a complex set of external and internal factors. Unsustainable extraction of these resources may also drive changes in diversity and species composition [7]. Consequently, it is important to assess variation in natural resource use and its concomitant effect on biodiversity using metrics that accurately reflect the utilitarian demands of rural forest-dwelling communities.

Predicting how local-scale human activities influence population- and community-level forest dynamics can be complex. One way to assess that process is by quantifying traditional uses of wild plants—the utilitarian species in the forests [8], [9]. These plants are essential for maintaining people's livelihoods and well-being, as they are ecosystem service providers (ESPs) [4], [10]. A clear understanding about the condition of provisioning services in particular, relies upon knowing both the resource use patterns of the people who are most reliant on those services, as well as the utility of the plants exploited by local people [11]. Our goal in this paper is to present a practical framework for quantifying the status of forest provisioning services by assessing the functional diversity and redundancy of utilitarian plant species (see below). Although our case study examines natural resource use in rainforests in southeastern Madagascar, the methods we describe are applicable to other environments and regions.

### Functional diversity and functional redundancy with utilitarian species

**Figure 1:**
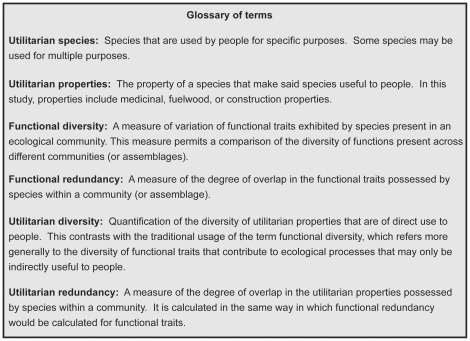
Glossary to distinguish between terms used in ecological studies on functional trait and those used throughout this manuscript.

Functional diversity (FD) measures the variation of traits for species present in an ecological community and provides a way to compare the diversity of species' roles between plant communities. In the ecological literature on plant functional diversity, commonly assessed traits include specific leaf area (SLA), wood density or height [12]. In contrast to metrics such as species diversity, FD accounts for the functional distinctness of an assemblage (e.g., plot or ecological community) of species rather than simply their taxonomy, and in some cases can provide greater explanatory power about ecosystem functions and services [13], [14], [15]. In this study, we calculate FD for an assemblage of plants using utilitarian properties (e.g., firewood, construction wood) instead of traits defined by ecological function. In this context, the term “utilitarian property” is analogous to functional traits; we refer to the diversity of these properties as “utilitarian diversity”, while the degree of overlap in properties is termed “utilitarian redundancy” (See Figure 1, Glossary of terms). Thus, even though utilitarian and ecological functional diversity are likely correlated, the degree to which they are related will be dependent on the number of functional traits and utilitarian properties considered. For instance, they may be perfectly correlated if all the utilitarian properties (e.g., construction) assigned to each species are directly associated with particular functional traits (e.g., high wood density), such that there is a one-to-one matching of trait and property. However, whether people's uses of wild plants are related to one or a suite of functional traits is unknown for most assemblages. Yet one would presume that forest-dependent communities would favor some species over others because of a certain utilitarian property that makes those species better for particular services.

A crucial component of FD and a corresponding feature of community assemblages is functional redundancy [16] (see also functional divergence in [17]). Functional redundancy indicates the degree of overlap in the traits of species within a community assemblage. If an assemblage has high species richness and shows little change in functional diversity as species are lost, then the species pool in the assemblage has high functional redundancy. That is to say, many species share similar traits, therefore the loss of a few species has little impact on FD. In contrast, an assemblage with low functional redundancy may exhibit considerable changes in FD with only minor changes in species richness [18]. From a human livelihoods perspective, low utilitarian redundancy could indicate how threatened certain services may be for local people. Ideally, for communities who depend on forests for many different purposes, plant communities would exhibit a balance between utilitarian diversity and redundancy.

Functional redundancy can be assessed by examining the relative change in FD and species richness across many community assemblages using field experiments or through simulations that model observed versus expected FD, given a particular species richness for an assemblage. In this study, we used the latter approach (see Methods for more details) [16], [19]. When assessed in this manner, if observed FD (or in our analyses, utilitarian diversity) is higher than expected, species are more functionally distinct from one another than expected by chance and this, in turn, indicates low redundancy. Conversely, environments that have lower than expected FD (utilitarian diversity) is an indication of high redundancy, such that species in a given plot are more similar than expected by chance.

In the context of human welfare and livelihood, ecological communities will exhibit higher or lower utilitarian redundancy as a consequence of many different extraction patterns. For instance, targeting particular species for unsustainable harvest could lead to plant communities with low utilitarian redundancy. Species may be targeted because of their availability in forests (abundance), people's preference [20], proximity to villages [7] or lack of available substitutes [9]. If such targeting resulted in only a few remaining species with a particular set of uses (e.g., only a small number of species used for construction remain), then those assemblages would have low utilitarian redundancy. Conversely, if the targeted species belong to the same group (i.e., offer the same service) and a remaining, relatively high number of species belong to another group of service providers, then that would result in high redundancy, since the remaining species would share the same properties. Therefore, a non-random distribution of utilitarian properties can result from the exploitation of natural resources, which could show both high and low redundancy. In reality, there will likely be multiple groups of service providers present in any one community, such that a combination of species will be used for medicine, fuelwood, construction and food. A service provider group (i.e., species that provide fuelwood) with high utilitarian redundancy would mean that more substitutes for a specific service are available, thus allowing local people to switch between preferred and alternative choices.

We used Ranomafana National Park (RNP), located in southeastern Madagascar, as a case study to further develop a framework for applying FD metrics to the study of natural resource use. We focused on three questions regarding availability of important service provider groups:

1. What is the utilitarian diversity and utilitarian redundancy within plant assemblages in RNP?
2. Which suite of anthropogenic and environmental variables most influence changes in utilitarian redundancy within community assemblages?
3. Which utilitarian property was most important within a given assemblage?

Given the rapid rate of forest loss in Madagascar [21] and prospective changes in availability of utilitarian species, we wanted to determine the utilitarian redundancy of tree assemblages surrounding rural subsistence communities in the study area. Considering that many subsistence communities in developing countries—such as Madagascar—are often reliant on forest resources for their livelihoods [2], [6], [22], we expected that the majority of assemblages would show low utilitarian redundancy. Regarding the second research question, we assessed variables associated with anthropogenic use patterns to determine the extent to which certain factors influenced utilitarian redundancy. We also included environmental variables unrelated to human use, because local-scale resource utilization in tropical forests is influenced by both anthropogenic and natural factors [23]. Finally, we expected that certain utilitarian plants would have been used more frequently than others. Given this disproportionate use, we hypothesized that resource extraction by local people would directly affect the identity of particular utilitarian properties, but expected these use patterns to be modulated by distance from villages. However, we did not have any *a priori* reasons to hypothesize that one utilitarian species would be more important than another.

## Materials and Methods

### Study site

**Figure 2:**
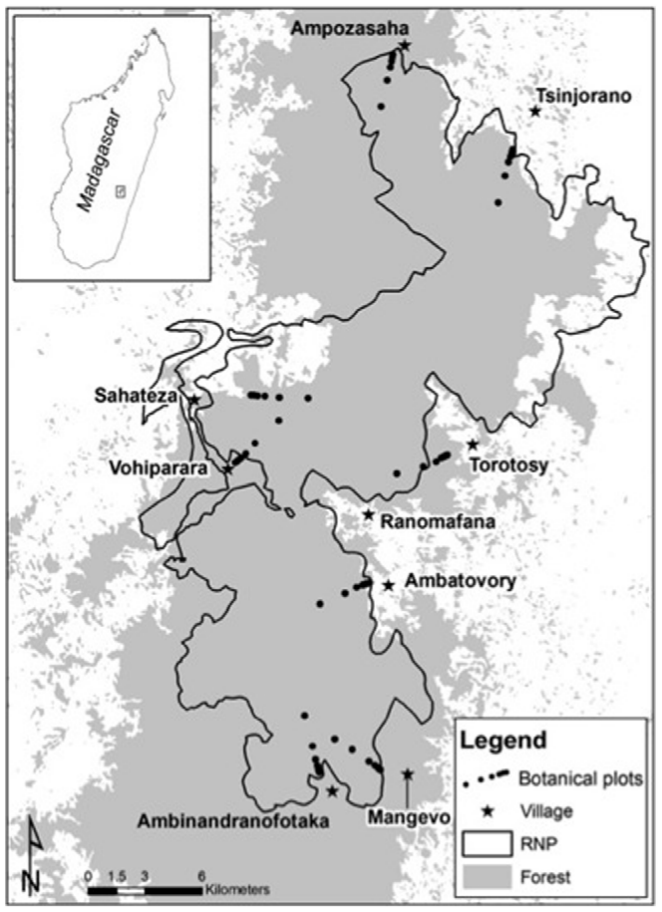
Site map showing the boundary for Ranomafana National Park (RNP). Filled circles indicate plots along the transect line moving from RNP's edge to its interior. The gray area represents forest limits.

Ranomafana National Park (RNP) is located between 47° 18′–47° 37′E and 21° 02′–21° 25 S (Figure 2). Ranomafana consists of 43,500 ha of continuous mid-altitude montane rainforest, with annual rainfall ranging from 1700–4300 mm. Since RNP was gazetted in 1991, local settlements have been restricted to the peripheral zones. These areas have been degraded through past and current human extraction, but some relict patches remain with relatively intact forest and mixed plantations [24]. Available figures estimate that 160 villages surround the forest area of RNP, with a total population of 27,000 within a 500 km^2^ area [24]; the population has likely increased in the past decade (C. Holmes, pers. comm.). Brooks et al. [25] and Brown et al. [26] provide a more detailed summary of the land use activities associated with RNP.

### Vegetation sampling

Vegetation sampling was carried out from October 2003–June 2005. Surveys were conducted in forests adjacent to eight villages on the periphery of RNP (Figure 2), corresponding to localities where human land use has been investigated in related research (see [25]). Seven plots measuring 40 m×40 m were established along a transect at each site, with a total sampling effort of 1.12 ha per site. Each plot was treated as a separate assemblage. The transects commenced 10 m from the forest edge and were oriented approximately perpendicular to the edge; plots were set at the following edge-interior distance intervals along the transect: 0 m, 100 m, 200 m, 400 m, 800 m, 1600 m, and 3200 m. Within each plot, we recorded the diameter at 1.3 m height (DBH) for all woody stems >10 cm DBH and identified these stems to species (scientific and vernacular) and family.

We collected evidence of local residents' use of the forests that could have influenced utilitarian properties within each plot. Evidence of the following activities were systematically collected: (1) number of felled trees; (2) number of damaged trees (e.g., bark stripped, branches cut); (3) number of human trails; and (4) amount of cattle dung. Trees categorized as “felled” were clearly cut down by people, as signs of this activity are persistent. The position of each village was recorded at its center; and the distance from each plot to the closest village was determined using ArcGIS 9.3. The mean canopy height and elevation of each plot was also taken. We also calculated the basal area for each assemblage using DBH.

### Utilitarian properties

Utilitarian properties for the species encountered in the vegetation survey were determined by two methods: (i) expert knowledge and (ii) household questionnaires. First, traditional ecological knowledge was recorded from local village experts about local people's uses of plant species, with construction and firewood species ranked according to preference. These species were classified according to six utilitarian properties (details below). Secondary data were gathered from villages surrounding another protected area, Manombo Special Reserve and Manombo Classified Forest, which is 175 km south of RNP [27]. We used data collected from Manombo's humid forests and concentrated only on those species that shared uses, local vernacular and scientific names with those found in RNP. The second approach used structured household questionnaires derived from a total of 247 households, to gather more detailed information about residents' consumption patterns for firewood and construction species, as well as preference for construction species. NKA sought and received verbal informed consent from all participants; however the data were analyzed anonymously. The ethics committee at Stony Brook University approved the consent procedure for collecting data on expert knowledge.

### Utiliarian diversity calculations

For our purposes, “functional diversity” refers to the diversity of utilitarian properties across species in a given ecological community. We used Petchey and Gaston's Functional Diversity (FD) [28] as a measure of utilitarian diversity [26]. FD is a dendrogram-based index of functional diversity. We used the Gower method to calculate multivariate distances between species because our trait data were of mixed types (continuous, ordinal, and binary) and unweighted pair-group method with arithmetic means (UPGMA) to create the functional dendrograms, because this clustering algorithm gave the highest cophenetic correlation with the original multivariate distances [29]. Species' uses of interest were as follows: construction (ordinal), firewood (ordinal), medicinal (binary), food (binary), wood for tools (binary), and wood for furniture (binary). Construction and firewood species were ranked into three preference categories based on their level of use by local residents: high, moderate and least preferred.

To distinguish whether an observed change in utilitarian diversity was simply a product of changing species richness (S) and to assess the level of utilitarian redundancy within service provider groups comprising these communities, we used a simulation approach to create a null distribution of utilitarian diversity values for the observed number of species. The goal of the null models was to detect if there were any non-random changes in species composition. Holding species richness constant for each of the 56 plots, we randomly selected species from the species pool (the total number of species in the study) to calculate a null utilitarian diversity for each community. We repeated this 5000 times to produce a distribution of null values and tested whether the observed utilitarian diversity for each community was significantly higher or lower than the expected utilitarian diversity values at α = 0.05. The “expected utilitarian FD” is the mean of the utilitarian diversity calculated from the 5000 null communities for each plot.

In an effort to determine which utilitarian property was most important, we sequentially removed one property from each simulation. We then calculated a new FD without that utilitarian property and compared it to the FD from the model that included all the properties (e.g., FD_all_ vs. FD_firewood_; FD_all_ vs. FD_construction_, with the property listed indicating removal from the model). The new FD value that had the largest decrease from the original FD was deemed the most important property. This analysis was done at the plot-level and we report values averaged across plots.

Utilitarian redundancy was assessed using the Index of Variance (IV) [30], which is the ratio of the observed to expected utilitarian diversity for each forest plot:
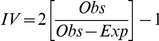
where *Obs* is the observed utilitarian diversity and Exp is the mean utilitarian diversity from the randomizations. The IV gives a continuous measurement of redundancy and a positive IV value indicates that observed utilitarian diversity is greater than expected utilitarian diversity while holding species richness constant (i.e., high IV correspond to low utilitarian redundancy).

### Generalized Linear Models (GLM)

We used Generalized Linear Models (GLM) to analyze the relationship between utilitarian redundancy (as reflected by the Index of Variance [IV] from the null model) and six predictor variables: basal area, number of felled trees, canopy height, distance from village, number of trails and elevation. We did not include the variables damaged trees nor cattle dung incidence, because of the overabundance of zero values for those variables. The GLM model used for these analyses comprised error terms approximated by Gaussian distribution and an identity link function.

The performance of the GLM was assessed using Akaike Information Criterion (AIC). We used two measures associated with the AIC to determine the optimal model given the data. The 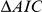 ranks alternative models according to their AIC values and the AIC for each model is rescaled based on the best model. Therefore, the 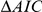 measured the fit of each model relative to the best model which has an 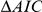 = 0. Small differences in 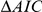 (<2) indicate convincing evidence for the model and values between 3–7 indicate less support [31]. The other measure used to assess model optimality was the Akaike weights, which indicated the probability that a given model was the best among the whole set of candidate models [31].

### Regression Trees

Identifying which utilitarian properties are associated with trees at the varying distances from village cannot be accomplished by using a metric like FD, which summarized utilitarian diversity across the community. We used a regression tree approach to test the association of utilitarian properties with tree size at different distances from the village. For a given distance from villages (across all sites) we applied regression trees, which recursively partition the predictor variables (the utilitarian properties) to explain the variation in the response variable, which was tree size, as measured by DBH. We used the sum of squares around group means to establish splitting criteria, selected optimal tree size by K-fold cross-validation, and evaluated model fit with Pearson correlation of predicted to observed DBH [32]. Therefore, the results of this approach showed which utilitarian properties were most closely associated with variation in tree size, depending on distance from village. All calculations and statistical analyses were carried out using the statistical program R [33]; the optimal GLM models were selected using MuMIn package version 0.13.17 [34].

## Results

### Utilitarian diversity and redundancy: null model approach

**Figure 3:**
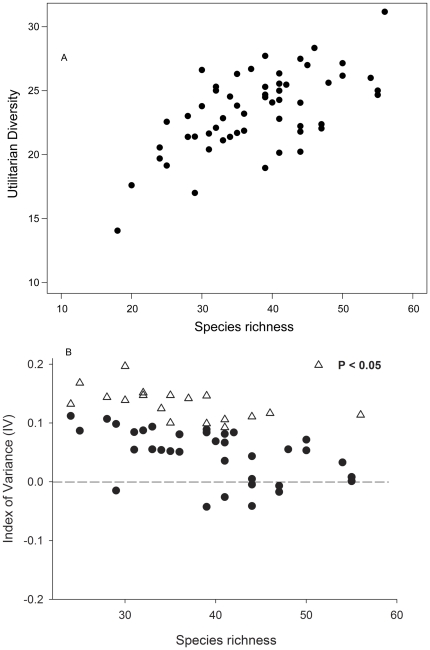
Scatterplots showing results from the utilitarian diversity and redundancy null model analyses. (**A**). Functional diversity plot showing observed utilitarian diversity versus species richness. (**B**). Index of variance (IV) values for the utilitarian diversity indices versus species richness. A positive IV value indicates that observed utilitarian diversity is greater than expected utilitarian diversity while holding species richness constant (i.e., it indicates low utilitarian redundancy). Triangles indicate plots that were significantly greater than expected at α = 0.05. *N* = 56.

The null model simulations assessed whether observed utilitarian diversity deviated significantly from that of the randomly assembled utilitarian species pools in RNP. Utilitarian redundancy increased positively with species richness, with indications that the initial non-saturating relationship was beginning to plateau at high species richness (Figure 3A). Eighteen of the 56 plots exhibited IV values significantly higher than expected by chance at α = 0.05, indicating low utilitarian redundancy (Figure 3B). None of the plots showed significantly lower than expected IV values, which would have indicated high utilitarian redundancy (Figure 3B). Although the remaining 38 plots did not exhibit significantly low or high IV values at the established α level, there is a notable trend of plots with low utilitarian redundancy (30 plots), with very few having high redundancy. Moreover, The null model removal simulations indicated that the most important utilitarian properties within each assemblage were construction and firewood (FD_all_ = 20.1 vs. FD_construction_ = 12.2 and FD_firewood_ = 13.7), while the least important were medicinal and wood for furniture (FD_medicinal_ = 19.2 and FD_furniture_ = 19.1).

**Figure 4:**
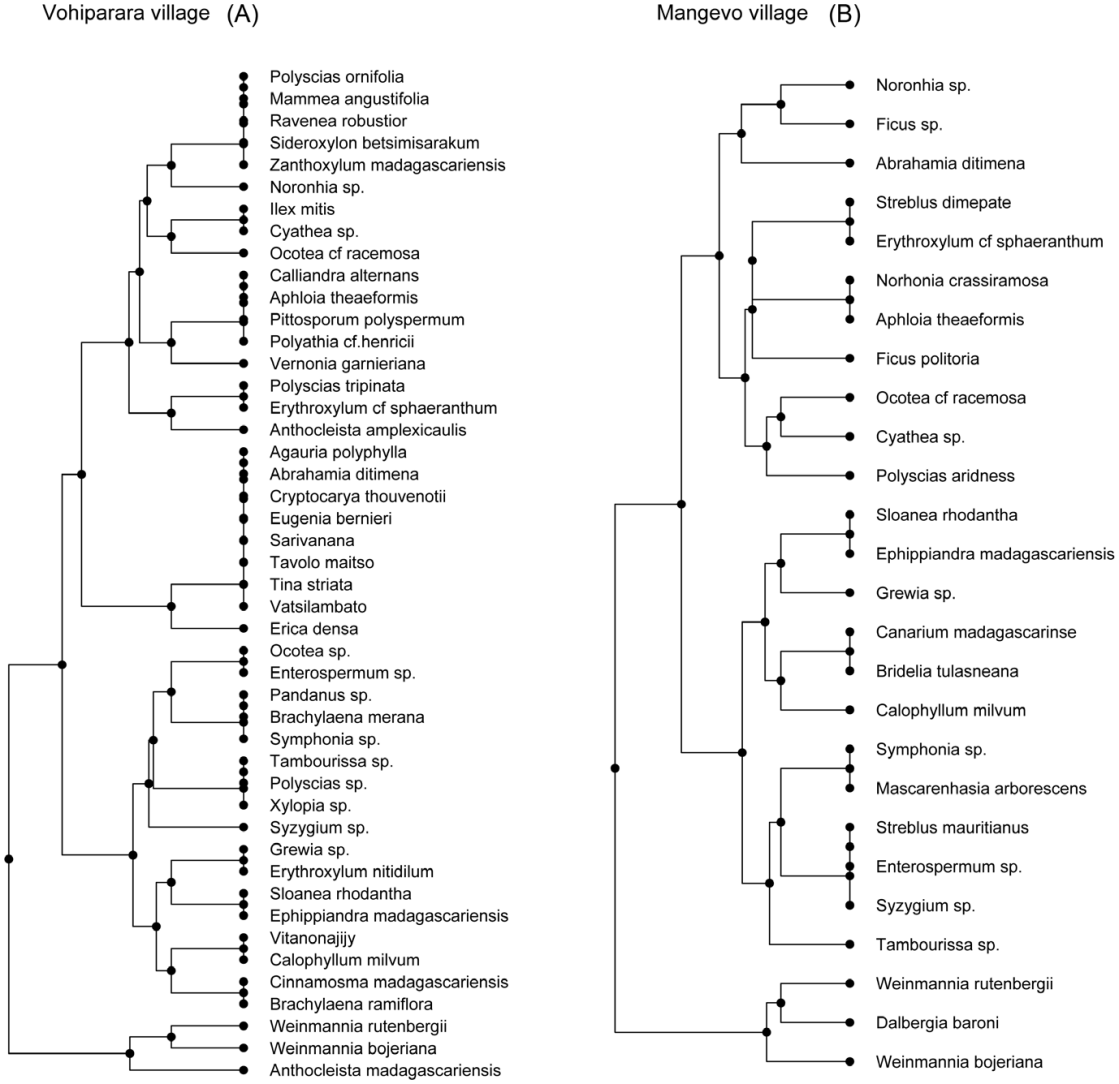
Dendrogram of the utilitarian relationships among plant species used for provisioning services for the villages of Vohiparara (4A) Mangevo (4B). The dendrogram was produced by hierarchical clustering by the UPGMA (Unweighted Pair Group Method with Arithmetic Mean) algorithm of the distance matrix, which was calculated based on species' uses. Horizontal distance represents separation in “utilitarian space”. Species richness for Vohiparara = 46 and Mangevo = 26; the observed utilitarian diversity for Vohiparara is 24.2 and Mangevo is 33.8; and IV for Vohiparara = 0.08 and IV for Mangevo = 0.03.

The functional dendrogram describes the relationship among the utilitarian species in the study (Figure 4). Examples are given for low and high utilitarian redundancy plots, Vohiparara (Figure 4A) and Mangevo (Figure 4B) respectively. Species on opposite ends of the dendrogram are most dissimilar in terms of utilitarian properties, while those in closest proximity have similar properties. For example, *Polyscias ornifolia* and *Mammea augustifolia* are both low preference construction species (Figure 4A). The other end of the dendrogram shows *Anthocleista madagascariensis* and *Weinmannia bojeriana*, which are highly preferred species used for both construction and firewood (Figure 4A). Each of those two species also has secondary uses, with the former used for medicine and the latter's fruit used as food. Consequently, both functional dendrograms illustrate the fact that many of these plants have multiple uses. The major difference between the dendrograms is that Mangevo, the high redundancy plot contains several sets of species with similar uses, indicating an elevated degree of utilitarian redundancy of utilitarian properties (Figure 4B). For instance, *Cryptocarya thouvenotii*, *Abrahamia ditimena*, *Diospyros* sp, *Eugenia emirnensis*, and *Homalium albiflorum* are all rated as moderately good firewood species. Consequently, even though Vohiparara village has higher species richness (S_Vohiparara_ = 46 vs. S_Mangevo_ = 26), Mangevo village exhibits higher redundancy (Figure 4).

### Influence of predictor variables: GLM analysis

The GLM consisted of six predictor variables and results are presented for the models with a 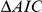 of <2. The AIC scores indicated that the best model fit included only basal area; two additional models (with basal area and distance, and with basal area and canopy height) were also a good fit to the data (Table 1). Thus, the 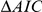 comparisons supported a strong basal area effect, since all the best-fit models, with the exception of one, included that variable as a predictor (Table 1). The variance explained was lower for the optimal model (*R* ^2^ = 0.091) than for the next top two models that included distance (*R^2^* = 0.118) and canopy (*R^2^* = 0.114). The model that explained the highest amount of variation included basal area, distance to village and canopy height (Table 1), although R^2^ values were relatively low for all models (suggesting there may be other important predictors).

**Table 1:** Summary of selected optimal Generalized Linear Models (GLM) fitted to utilitarian redundancy (assessed as Index of Variance, IV) and six predictor variables.

Models	*df*	AIC	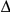 AIC	Akaike weight	R^2^
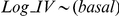	54	−172.8	0.00	0.114	0.091
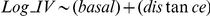	53	−172.4	0.68	0.081	0.118
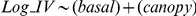	53	−172.2	0.91	0.073	0.114
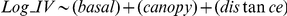	52	−171.5	2.01	0.042	0.134
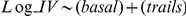	53	−170.9	2.22	0.038	0.093
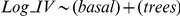	53	−170.8	2.32	0.036	0.091
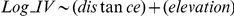	53	−171.0	2.32	0.036	0.091

*The full model  =* 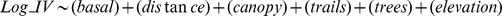. Deviance explained = 14.8% (*n = 55*), residual deviance = 12.7% (*n = 49*). AIC is the Akaike Information Criterion; see text for details on 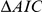 and Akaike weights. Coefficients: intercept = 1.24×10^−1^; basal area = −5.81×10^−4^; distance = −8.26×10^−6^; canopy = −2.51×10^−3^; trail = 4.87×10^−4^; trees cut = −3.51×10^−4^; elevation = 2.55×10^−5^.

**Figure 5:**
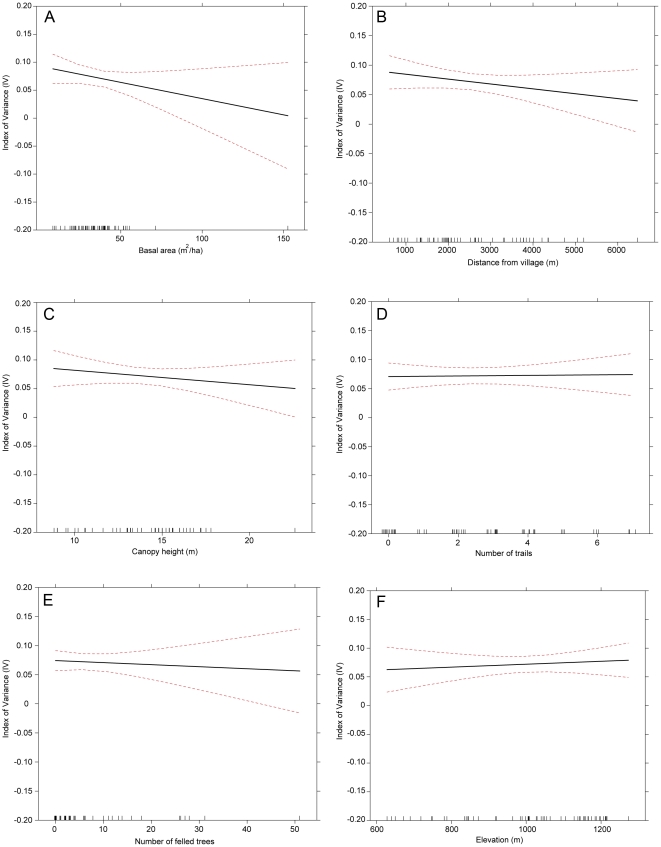
The response of the Index of Variance (IV) to variables included in the Generalized Liner Model (GLM). The response of IV to: **A.** basal area (m2/ha); **B.** distance from village (m); **C.** canopy height (m); **D.** number of trails; **E.** number of felled trees; and **F.** elevation (m). Solid black lines indicate response and dashed lines indicate 95% confidence intervals for the mean response.

Moreover, there was an inverse relationship between IV and basal area, indicating that utilitarian redundancy was lowest in assemblages with small basal area (Figure 5A). The variables included in the other two top models exhibited similar inverse relationships. Index of variance decreased with increasing distance from village, suggesting that assemblages closer to villages were more likely to have low utilitarian redundancy than those farther away (Figure 5B). Also, IV values decreased with increasing canopy height, which indicated that lower utilitarian redundancy occurred in short statured assemblages (Figure 5C). The models that included number of trails, felled trees and elevation did not exhibit any consistent patterns (Figures D–F).

### Regression tree analysis

**Figure 6:**
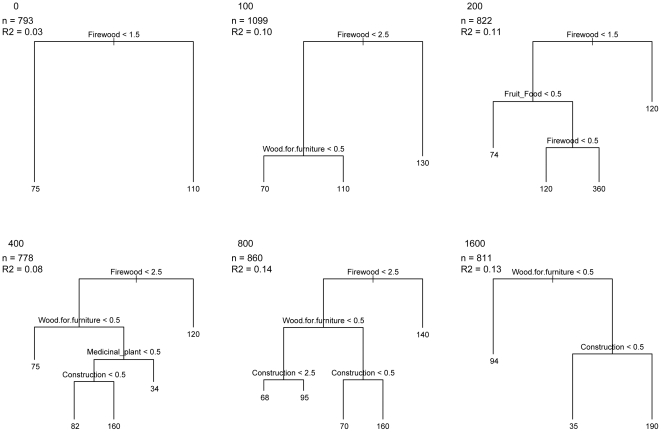
Best-fit regression trees relating utilitarian properties to tree size (as measured by diameter at breast height, DBH) at increasing distances from villages. Results are shown across all species, for all sites. Split labels show the criteria for splitting the right-hand branch from the left-hand branch. Tip labels represent mean DBH of the individuals at the tip. For example, at 0-m from village (A), the optimal regression tree identified species used for firewood and rated 2 (good) or 3 (very good) as most associated with large-girth individuals (with a mean DBH of 110 cm). No other utilitarian property was associated with tree size at this location.

Firewood was the primary splitting variable for all distances, with the exception of those plots far from the forest edge (Figure 6). Moreover, this primary split was most associated with large trees. Specifically, at the forest edge, the sole split was firewood (Figure 6A; *R* = 0.03), while for 100-m from edge, variables for splitting included firewood and wood for furniture, with the latter most associated with larger trees (Figure 6B; *R* = 0.10). At 200-m, a secondary splitting variable was associated with large trees used for food and another split based on firewood (Figure 6C; *R* = 0.11). For distances 400-m and 800-m from the forest edge, there was a secondary split based on wood used for furniture; with the 400-m distance also split according to trees used for medicine and then large girthed individuals used for construction (Figure 6D; *R* = 0.08) and at 800-m there was only a split for large trees associated with construction (Figure 6E; *R* = 0.14). The strongest predictor of utilitarian properties associated with tree size at 1600-m was large individuals used to make furniture and also for construction (Figure 6F; *R* = 0.13).

## Discussion

### Utilitarian diversity and redundancy

Our goal in assessing utilitarian diversity and redundancy was to place the ecological underpinnings of natural resource use within a social context by focusing primarily on provisioning services that were important for rural, subsistence people in Madagascar. Our results showed that many areas had low utilitarian redundancy, which implied that these assemblages have a moderate to high risk of losing the diversity of utilitarian properties provided by forests. These results also highlight the importance of two utilitarian properties—construction and firewood—for local residents. For many rural communities, there is a complete dependence on forest services for firewood and construction material. Similarly, NTFPs from forests often constitute a crucial aspect of people's livelihoods, especially in times of agricultural shortfalls, droughts, pestilence or other emergencies [35]. Consequently, this issue of low redundancy is important, because it suggests that few alternative species could be used as substitutes for more vital services, such as construction. If the differences between low and high utilitarian redundancy forest assemblages is being driven by land use change, then residents' livelihoods surrounding forests with low redundancy may become increasingly challenging, since minor losses in species richness would result in substantial reductions in utilitarian diversity.

In light of these results, one of the goals for conservation, particularly when dealing with rural, subsistence people, should be to enhance or maintain high functional redundancy within ESPs or each service provider group. Moreover, at a community or landscape scale at which multiple service provider groups may be present to supply a range of services (i.e., fuelwood, medicine, construction materials), it is important to maintain a diversity of service provider groups, reflected by high utilitarian diversity. Within each service provider group, it would be important to maintain high utilitarian redundancy, so that the needs of people can be sustained throughout time by ensuring multiple species are available to provide that service in the case that one or several species decline.

One of the advantages of analyzing utilitarian diversity and redundancy is that areas at risk of losing utilitarian species can be identified and be used to prioritize areas for conservation action in conjunction with other priority setting conservation schemes. In this respect, habitats that fall within the significantly lower than expected categories can be directly targeted for either participatory forestry management with local communities or habitat restoration projects that focus on native utilitarian species. Similarly, conservation efforts to maintain certain ecosystem service providers would benefit the livelihood of local residents. For instance, construction and firewood were identified as the two most important utilitarian properties and contributed most to changes in utilitarian diversity. Therefore, species associated with both of these provisioning services are important resources for villagers surrounding RNP and could be specifically targeted for sustainable management programs.

### Factors driving variation in utilitarian redundancy

The observed relationship between index of variance (IV) and the three most important predictor variables indicated that there was a strong basal area effect that was inversely related to utilitarian redundancy. This relationship suggested that large-girthed trees were associated with fewer utilitarian properties. We suspect that specific utilitarian properties—and consequently the availability of alternative resources—were scarce or absent from assemblages with large-girthed species, because local residents have harvested many of the small-to-medium sized utilitarian trees. Such trees would most likely be used for construction, particularly medium-sized planks used predominantly for flooring and in some cases to reinforce walls of houses [27]. With regard to firewood, results from the regression tree showed that at almost all distances from the forest edge, firewood was the utilitarian property most useful in distinguishing between large and small trees. Either “good for firewood” is consistent with “grows big and fast”, or local residents are leaving large trees even though they are good for firewood. We suspect the latter, since circumstances wherein the entire tree is harvested is most likely restricted to small girthed trees. We continue to research this question, as little is known about the functional traits associated with utilitarian species and how those traits are related to human use.

Our analyses also showed that village proximity plays an important role in the extraction rate of natural resources. Studies in tropical forests, including Madagascar, demonstrated a similar pattern between human impact and village proxmity: use of forest resources declines with increasing distance from forest boundary [7], [36], [37]. One of the implications of this result is that assemblages with high diversity of utilitarian properties are found increasingly farther from villages, and local residents may need to travel greater distances to access certain provisioning services.

Overall, our analyses suggest that variation in utilitarian redundancy was most likely a result of local residents harvesting utilitarian species from forests associated with villages surrounding RNP. Resource use that could have led to this pattern occurs if residents overharvest species with certain uses, leading to a steady decrease in particular utilitarian properties. For instance, a spate of construction in the RNP region due to large numbers of new migrant households that have cleared lands between 1989–2003 [25] would result in selective harvesting of construction species. An influx of migrants would also increase pressure on firewood species and natural resource use in general. These results are supported by other studies which show that variability in biodiversity and forest structure can be partly modulated by the magnitude and intensity of resource extraction by subsistence communities [22], [38]. Our findings illustrate the connection between local residents' use of provisioning services and the associated decrease in the redundancy and diversity of utilitarian properties, which gives a direct assessment about whether forests would be able to provide a buffer against rapid declines in the availability of certain natural resources.

### Important considerations regarding utilitarian diversity and redundancy

It is important to note a few further caveats, however. First, this study defined species by their utility to local people (i.e., utilitarian properties) rather than their ecological functional trait values, as is traditionally the case when defining functional diversity [28]. Additionally, careful consideration should be given to the choice of “properties” used to develop utilitarian diversity. We focused on utilitarian properties, but including species used for cultural and other services could be important for developing conservation opportunities that integrate research approaches from different disciplines, while also emphasizing the complex interactions between social and ecological systems [39]. Furthermore, functional diversity metrics are affected by the number and choice of traits used. With respect to these analyses, incorporating a greater number of utilitarian properties, especially if some are not relevant to the question at hand, may artificially reduce utilitarian redundancy by making species appear to be more different from each other than they are in practice [16].

Ecological processes unrelated to harvesting of natural resources, such as limiting similarity or environmental filtering can regulate community assembly and species composition. Limiting similarity occurs when species with similar traits cannot coexist within the same habitat, particularly at increasing densities at relatively small spatial scales [40], [41]. Environmental filtering restricts community membership to species that possess traits that enable their growth and survival in the particular environment [41]. Comparisons between these competing hypotheses can be explored by examining different functional trait distributions, similar to the analyses done here. For instance, greater observed than expected functional diversity can be viewed as evidence for limiting similarity among species, while lower observed than expected functional diversity can be viewed as evidence for environmental filtering [42]. For our purposes, we did not directly address these competing hypotheses; instead, we focused on describing utilitarian diversity and redundancy. We recognize that such ecological processes can produce patterns in diversity and redundancy similar to those influenced by anthropogenic activities, and that the analytical techniques employed here cannot adequately resolve whether natural or anthropogenic drivers are controlling these patterns. Consequently, our results were interpreted cautiously.

Furthermore, whether local residents are affected by low utilitarian redundancy will depend on the extent to which they integrate collection of wood and NTFPs with subsistence farming to supplement their livelihoods [43]. Subsistence societies' reliance on natural resources may vary from village to village and household to household depending on livelihood options [6] and might be affected differently by a decrease in utilitarian diversity. For instance, households around RNP that rely on crayfish (*Astacoides* sp.) to supplement agricultural shortfalls, through both consumption and trade [44], might be affected less by a decrease in the utilitarian diversity of tree assemblages. Also, the degree of household vulnerability due to low utilitarian redundancy will depend directly on the uses of the species that are lost. For example, low redundancy for construction species might be a cause for concern, particularly if there is increased frequency with which people construct (or reconstruct) homes (e.g., due to a high prevalence of cyclones); in contrast, households may not need to be as selective when choosing firewood, where a wide variety of species may be used. As a further note, it also should be pointed out that utilitarian properties are defined by presently observed use patterns, and it is possible that other forest species could begin to take these functions under conditions of scarcity.

### Conclusion

Overall, the advantage of the approach taken in this study is that it presents a quantitative method for integrating ecological, social and cultural systems to address the condition of provisioning ecosystem services. Equally important, this approach translates an analytical method from the ecological literature and extends it to an important applied, socio-ecological question about natural resource use, allowing for a potential exchange of information between researchers and resource managers.
